# Vaccines and myasthenia gravis: a comprehensive review and retrospective study of SARS-CoV-2 vaccination in a large cohort of myasthenic patients

**DOI:** 10.1007/s00415-022-11140-9

**Published:** 2022-05-03

**Authors:** Giulio Sansone, Domenico Marco Bonifati

**Affiliations:** 1 Department of Neuroscience, University of Padova, Via Nicolò Giustiniani, 5, 35128 Padua, PD Italy; 2 Ca’foncello Hospital, Treviso, Italy

**Keywords:** Myasthenia gravis, Vaccines, SARS-CoV-2, Review

## Abstract

**Introduction:**

Myasthenia gravis (MG) is an autoimmune disease, for which the risk of exacerbation after vaccines is debated. The aim of this study is to review the available literature concerning safety and efficacy of vaccines in MG. In addition, we also conducted a retrospective research of MG exacerbations and new onset MG after anti-SARS-CoV-2 vaccination in a large cohort of patients.

**Methods:**

A study of the available literature regarding vaccines and MG was carried out through research in the online database “Pubmed”. We also retrospectively collected data from 80 MG patients, who were followed at the Treviso Hospital and completed an anti-SARS-CoV-2 vaccination cycle. For each patient, we recorded MG exacerbations between first and second doses and within a window period of 1 day – 6 weeks after the second dose.

**Results:**

We found 26 relevant articles about influenza, SARS-CoV-2 and other vaccines. No clear associations between most vaccines and MG exacerbations were found. Moreover, cases of new onset post-vaccine MG are mostly anecdotal, except for Japanese encephalitis virus vaccine. Concerning our cohort, 4/80 (5%) MG patients experienced an exacerbation within the post-vaccine window period. In addition, we report a case of new onset post-vaccine MG.

**Discussion:**

Inactivated and subunit vaccines are safe and effective in MG. Although some of them, such as anti-SARS-CoV-2 vaccine, might uncommonly cause MG exacerbations, data from our review suggest that benefits still outweigh by far the potential risks, thus they should be recommended to these patients. Nevertheless, large prospective studies are needed for further investigations.

## Introduction

Myasthenia gravis (MG) is a chronic autoimmune disease affecting the neuromuscular junction. From an epidemiological point of view, its prevalence is 100–200 cases/million people, whereas two age-related peaks in its incidence have been described: between 20 and 30 years in females and between 50 and 70 years in males. It is the prototype of antibody-mediated autoimmune disorders: the disease is caused by circulating antibodies against antigens located in the post-synaptic membrane of the neuromuscular junction. In about 85% of cases, the antigen is the nicotinic acetylcholine receptor (AChR), while antibodies against other proteins, such as anti-MuSK or anti-LRP4, are detected in a smaller percentage of cases [1].

Clinically, it is characterised by fatigability and fluctuating weakness of skeletal muscles. Such symptoms typically worsen in the evening and after physical exercise, while they improve with rest. The most frequently involved skeletal muscle groups are the ocular ones, even though all muscle groups can be involved, thus potentially causing fatigability in all four limbs and difficulties in the activities of daily living. Bulbar muscles (chewing, swallowing and speaking), facial and lingual districts are also frequently involved. In severe cases, respiratory muscles involvement and respiratory failure may occur. The main clinical phenotypes are the following: ocular, early onset (EOMG), late onset (LOMG), anti-MuSK antibody-associated, seronegative and thymoma-associated. Symptomatic treatments include acetylcholinesterase inhibitors such as pyridostigmine. Being an immune-mediated disease, its aetiological therapy is based upon corticosteroids and immunosuppressive drugs [1, 2].

The most severe and life-threatening complications of MG are bulbar exacerbations and myasthenic crises. In the latter cases, the clinical picture worsens significantly, leading to respiratory failure. The main risk factors for exacerbations and crises are infections, particularly respiratory ones, many medications, including sedatives and those that depress neuromuscular transmission, and insufficient or inappropriate therapy. Stressful factors such as bereavement, psychological or physical traumas can also trigger a myasthenic crisis [3]. In these cases, treatment is based upon plasma exchange (PE) or intravenous immunoglobulins (IVIg) [1].

The issue of vaccinations in patients affected by immune-mediated diseases has frequently raised concerns and speculations of possible causal relationships between certain vaccines and disease onset.

or exacerbation. For example, there are by now many articles and reviews reporting the risk of Guillain-Barré syndrome (GBS) following influenza vaccination[4, 5]; in particular, a metanalysis has confirmed the evidence of such a slight, yet significant risk [6]. Regarding multiple sclerosis (MS), several case reports and articles have been published; however, a review published in 2017 [7] highlights the absence of association between many vaccines and MS onset or exacerbation, except H1N1[8] and yellow fever[9] vaccinations, where further studies are needed to establish a potential causal relationship.

On the other hand, doubts about the efficacy of vaccines in a population of patients undergoing immunosuppressive therapies might arise. As a matter of fact, there are specific guidelines for vaccinations in such patients[10] but the risk/benefit ratio in each single disease and patient is not straightforward.

According to Coombs and Gell's classification[11], MG is a disease caused by a type-II hypersensitivity reaction and, as such, the production of pathogenic antibodies might precede clinical manifestations by years. For such diseases, it has been postulated that vaccines might respectively exacerbate or “unveil” manifest and subclinical pre-existing diseases, through non-specific systemic immune-stimulation, rather than triggering a de novo disease [12].

Lastly, regarding the relationships between vaccines and MG, there are also various experimental studies investigating the potential use of therapeutic vaccines or peptides against MG [13–17]. However, these issues will not be covered by the present review, as this subject ought to be deemed as experimental therapy for MG.

### Scope of the study

In this review we have searched the available literature, with the aim to answer to the following questions:

1. Are vaccines safe in myasthenia gravis and are there any risks of myasthenia onset or exacerbation?
2. Are vaccines effective in myasthenic patients with or without immunosuppressive therapies?

## Methods

A study of the relevant literature on the subject was carried out through a thorough search in the online database “PubMed”, using the criteria shown in Table 1, and by reference mining.

**Table 1:** MeSH terms used to research published articles in PubMed

MeSH terms used
[vaccination] and [myasthenia gravis]
[vaccination] and [MG]
[vaccines] and [myasthenia gravis]; [vaccines] and [MG]

138 articles were found applying the abovementioned search criteria (the last research was carried out on 23^rd^ January 2022). Articles not related to vaccination and MG were discarded, thus resulting in 29 relevant articles: 11 observational-descriptive, case–control or longitudinal studies, 16 case series or case reports and 2 reviews.

Furthermore, regarding anti-SARS-CoV-2 vaccination, we also retrospectively collected data from a large cohort of patients followed in our centre. The inclusion criteria were the following:

- MG patients who attended the myasthenic outpatient clinic at the Treviso Hospital from January 2021 to December 2021
- Completion of an anti-SARS-CoV-2 vaccination cycle (two doses)

For each patient, we considered the following variables:

- Sex
- Age
- Type of vaccination
- Time between last vaccine dose and MG exacerbation
- Presence of MG exacerbation within the window period of 1 day – 6 weeks after the second dose of SARS-CoV-2 vaccination, in accordance to the recently published WHO- and Bradley Hill-based AEFI classification system for neurological and neuropsychiatric diseases [18]. MG exacerbations between first and second doses were also considered.
  We divided our MG patients in the following subgroups:
- Group 1: stable disease during the window period, after the second vaccine dose
- Group 2: patients treated with plasma-exchange on a regular basis and without significant modification of disease activity after vaccination
- Group 3: development of MG exacerbation. Both mild and severe exacerbations were included, namely those requiring steroid/immunosuppressant introduction/dose increase or plasma-exchange/IVIg, respectively, between first and second doses and within the window period of 1 day – 6 weeks after the second vaccine dose

We also searched for new onset post-vaccine MG, using the same window period.

## Results

Results and evidence from the literature for each type of vaccine are summarised below.

### Influenza and H1N1 virus

An internet-based survey among 184 American neurologists, who followed 6465 MG, 2313 chronic immune-mediated demyelinating polyneuropathy (CIDP) and 1907 Guillain-Barré syndrome (GBS), showed that the influenza vaccine was recommended by 82.6%, 58.8% and 42.3% of the respondent neurologists for the three diseases, respectively. More experienced respondents (> 10 years of practice) tended to be more conservative in recommending the influenza vaccine to MG patients. A history of exacerbation following the influenza vaccine was regarded as the most important factor influencing vaccine recommendation for MG and CIDP [19].

A self-matched, population-based, retrospective study aimed at exploring the temporal association between influenza vaccination and myasthenic exacerbation among MG patients. Hospitalisations occurred in 513 MG patients out of 3667 within the observation period of 42 weeks following the administration of influenza vaccine. Those that occurred in the first 6 weeks (78) were compared to those that occurred in the control period (290), namely the 4-7th sets of 6 weeks. Relative incidence of hospitalization due to MG exacerbation during the risk interval compared with the control interval was 0.84 (95% confidence interval 0.63–1.06), indicating no increased risk of hospitalization for an exacerbation of myasthenia following influenza vaccination. Older patients had lower risk for MG in the immediate post-vaccination period, compared to younger patients, yet such finding was not statistically significant [20].

Another study was carried out as a survey through a questionnaire, with a retrospective longitudinal design. A total of 74 MG patients were included: 32 did not receive any vaccine, 4 H1N1 alone, 18 only seasonal influenza and 20 received both vaccines. For each group, the follow-up was at least 8 weeks after influenza vaccine administration and both significant and mild exacerbation were recorded. No exacerbations were found and only 3/24 patients who received H1N1 vaccine reported non-specific illnesses. Interestingly, the authors found that the most frequent reported causes of patient non-compliance to vaccinations were: fear of non-myasthenic side effects (42.6%), fear of a myasthenic exacerbation (31.5%), treating physician not recommending the vaccinations (14.8%) [21].

A multicentre survey involved a recall-based questionnaire administered to the included subjects during their first routine follow-up. A total of 258 MG patients were included, 133 of which had received an influenza vaccination and 121 had experienced either a common cold (96) or an influenza-like illness/ILI (25). MG exacerbation occurred in 40% of patients after ILI, in 15.6% of common colds, whereas it was reported only by 1.5% of patients who underwent influenza vaccination. The difference between exacerbation after ILI and after common cold was indeed significant (*p* = 0.006) [22]. Regarding vaccine efficacy, influenza vaccination did not confer protection for ILI since 11 (8.3%) of the 133 vaccinated patients had ILI compared with 14 (11.2%) of the unvaccinated patients.

A single-centre, prospective, double blind randomised, placebo-controlled study evaluated the efficacy and safety of influenza vaccination in MG patients with anti-AchR antibodies. The study included 47 anti-AchR antibody positive MG patients, furtherly subdivided into 29 patients with immunosuppressive therapy (IM +), 18 patients without (IM-) and 47 healthy controls. All of the aforementioned received either an influenza vaccination (H1N1, H3N2 and B-strain) or placebo, in a double-blinded fashion. The results showed no significant change of anti-AchR antibodies titres between IM + and IM- after influenza vaccination. No change in anti-AchR antibody titres was observed 4 weeks after influenza vaccination [23]. Post-vaccination seroprotection was similar between healthy controls, IM + and IM- and between thymectomised and non-thymectomised patients [23].

A single-centre, double-blind, randomised, placebo-controlled trial investigated the serological and clinical course of MG over 12 weeks after seasonal influenza vaccination. Sixty-two patients were included in the study. No significant difference in anti-AchR antibody titres between the two groups was found. The number of adverse events (74) was comparable between groups and the most common was the presence of flu-like symptoms. Adverse events occurred in 61.3% in the verum group and 41.9% in the placebo group, yet the difference was not statistically significant [24].

A case report showed the case of a 58-year-old woman, who developed bulbar MG 5 days after undergoing trivalent inactivated influenza vaccine. In particular, exclusive laryngeal involvement presenting with mild dysphagia and severe dysarthria, was reported. The patient was treated with pyridostigmine and steroid therapy, with significant symptoms attenuation after 2 weeks. The follow-up conducted 6 months after discharge showed complete remission of symptoms. [25]

### HBV

A case series reported two cases of post-HBV vaccine MG [26, 27]. The first case was characterised by a generalised myasthenic exacerbation occurring in the month following the second dose of HBV vaccine [26]. In the second case, onset of MG occurred 1 week after the first dose in an asthmatic patient. The clinical presentation included tetraparesis, dysphonia and dysphagia; anti-AchR antibodies were positive [26].

Furthermore, other two case reports regarding asthmatic patients who developed MG after anti-HBV vaccination have been described[27–29]; anti-AchR antibodies were not measured and negative, respectively. In particular, in one case HBV vaccination was administered, 3 days earlier, by general anaesthesia, induced and maintained with the following anaesthetics: fentanyl, thiopental, atracurium, and isoflurane. The patient developed the first symptoms 1 month after receiving the vaccine dose, afterwards ensued by generalisation. The stabilization of this patient’s MG entailed plasma exchanges and immunosuppressive drugs[28]. The second patient, instead, developed a myasthenic crisis 4 years after onset, and required ventilator care and IVIG [29].

Nonetheless, a review indicated as extremely rare the risk of developing myasthenia gravis after HBV vaccination (only one case reported in the cited study) [30, 31]. Among all cases of post-HBV vaccine MG presented above, only the first one was caused by a recombinant inactivated HBV vaccine, whereas all the others occurred after obsolete HBV vaccines derived from human donor plasma.

### HPV

We found a single case report regarding the possible relationships between myasthenia gravis and the nine-valent HPV vaccine, based on inactivated viral-like particles. The authors described the case of a 23-year-old woman who presented with a life-threatening form of MG, characterised by both ocular and bulbar involvement. Symptoms occurred on the third day after the second HPV vaccine administration, causing the patient to be transferred to the intensive care unit. Signs and symptoms resolved completely after 4 weeks, without recurrence after 5 months [32].

### Japanese encephalitis virus

One study investigated the possible causes of the high incidence of childhood onset MG (CMG) in China [33]. In the retrospective part of the study, more than 50% of the 4219 MG cases were CMG. In the prospective part, they longitudinally followed 104 cases of CMG and 100 healthy controls, who received the usual vaccinations included in the Chinese immunisation program, including live-attenuated Japanese encephalitis virus vaccination (LA-JEV). Mice injected with such vaccines were also studied to investigate the roles in CMG pathogenesis. The study found an increase of anti-AchR antibodies in both children and mice injected with LA-JEV, but not with the inactivated anti-JA vaccine. Furthermore, the same mice had a reduction of AchR density at NMJs, together with a decreased muscle strength and positive repetitive nerve stimulation. They also found a peptide of LA-JEV structurally similar to the AchR-alpha subunit and immunisation with a synthesised protein containing such peptide induced a MG-like phenotype in mice [33].

### Bacillus Calmette-Guerin

To our knowledge, there are only two case reports in literature that regard the association between bacillus Calmette-Guerin (BCG) and MG. In the first one, a 69-year-old man with bladder cancer was treated with intravesical BCG and developed ocular anti-AchR antibody positive MG 4 days after the completion of the 6-week treatment. The symptoms disappeared completely 12 days after the first dose of steroids [34].

In the second case report, another 69-year-old man with bladder cancer became affected by seropositive generalised MG within 1 month of intravesical BCG treatment. He was treated with steroids, IVIg and underwent thymectomy; 17 months after BCG treatment, the patient only showed minimal disease manifestations [35].

### Tetanus

A placebo-controlled, longitudinal study aimed at investigating the immune response to and safety of tetanus revaccination in 65 MG and LEMS patients, using 23 distinct placebo-receiving anti-AchR antibody positive MG patients and a historic healthy control group (revaccinated with tetanus toxoid). Although patients undergoing immunosuppressive therapies had significantly lower pre- and post-vaccine anti-tetanus titres compared to controls (*p* < 0.01; *p* = 0.02), their immune response was indeed still significant and protective [36]. In addition, although 5/65 patients did not reach the required increase of antibody titre to be classified as responders, all patients were considered protected against tetanus, according to WHO guidelines[37], and anti-AChR, MuSK or VGCC titres remained unchanged 4 weeks after vaccination; moreover, no myasthenic exacerbations were registered [36].

A Hungarian case–control study compared serum concentrations of tetanus-antitoxoid IgG between 158 MG patients, 279 systemic lupus erythematosus (SLE) patients and 208 healthy control subjects. In the group of patients with myasthenia gravis, there was no significant difference between patients with or without detectable anti-acetylcholine-receptor antibodies in the titres of tetanus-antitoxoid IgG. In all three groups, titres decreased only in the elderly subjects (> 60 years). There were no significant differences among the groups in the age-related changes [38].

### Diphtheria

The same case–control study described just above showed that in all three groups diphtheria-antitoxin IgG titre decreased significantly (*p* < 0.001) with age; furthermore, there were no significant differences among the groups in the age-related changes. Only a slight difference was detected between controls and MG patients’ anti-diphtheria titres (0.14 [0.06–0.33] vs 0.10 [0.04–0.29], *p* = 0.02). In the group of patients with myasthenia gravis, there was no significant difference between patients with or without detectable anti-acetylcholine-receptor antibodies in the titres of diphtheria-antitoxin antibodies (*p* = 0.196) [38].

### Neisseria meningitidis (meningococcus)

A retrospective observational study was conducted on Kaiser Permanente Southern California in 2–10-year-old children who received the MenACWY-CRM vaccine against Neisseria meningitidis. Preliminary results showed one case of post-vaccine myasthenia gravis, subsequently refuted after physician investigator review [39].

### Staphylococcus pneumoniae (pneumococcus)

We found a longitudinal study, aimed at investigating the effects of MG therapies on the immune response to the 23 polysaccharidic antigen pneumococcal vaccine. Twenty-five myasthenic patients were classified according to their MG treatment and their pre- and post-immunisation titres were compared to those of 11 control patients. The only side effects reported were minor soreness and inflammation at the injection site. All MG patients remained clinically stable, without exacerbations of muscle weakness in the post-immunisation period [40]. Post-immunization titres were not significantly different between MG and control patients. MG patients receiving no immunotherapy or receiving prednisone had pre- and post-immunization titres similar to those of control patients. The most significant finding was that MG patients receiving prednisone and chronic plasmapheresis had higher pre-immunization titres than did other patient groups and had significantly higher postimmunization titres against multiple pneumococcal serogroups than other MG subgroups. Such increase of the antibody response, presumably induced by plasmapheresis, was abolished by the concomitant administration of azathioprine. MG patients receiving no immunotherapy or receiving prednisone had pre- and post-immunization titres similar to those of control patients [40].

#### SARS-CoV-2

To date, there is still a lack of studies assessing the safety and efficacy of anti-SARS-CoV-2 vaccines in MG patients.

In a retrospective single-centre case series, the authors reviewed data from 22 known MG patients that were vaccinated against SARS-CoV-2 (21 with inactivated vaccine, 1 with recombinant subunit vaccine). Patients were classified as stable in case of resolution of symptoms for at least 1 month before the vaccination; in other cases, MGFA classification was used. The main observation outcome was worsening of MG symptoms within 4 weeks of vaccination, defined by a subjectively reported increase (> / = 2 points) in MG-Activity of Daily Living (MG-ADL) score, compared to the pre-vaccination status. Only two patients experienced mild and rapidly-resolving worsening of MG symptoms. In particular, one patient reported mild neck muscle weakness 7 days after the first dose of recombinant subunit vaccine, whereas the second patient reported neck and limb weakness 20 days after the first dose of inactivated vaccine. In both cases, quick resolution of symptoms after pyridostigmine dose increase occurred [41].

In a case report, a 77-year-old man, with a 5-year history of MG, experienced new-onset dysphagia, tachypnea and hypoxia after receiving the first and second doses of anti-SARS-CoV-2 Moderna vaccine, 5 weeks and 1 week earlier, respectively. On admission, he was diagnosed with MG exacerbation, in the absence of a triggering infection, and was treated with pyridostigmine and IVIg, yet developed a second crisis after 6 days. After treatment intensification, ICU transfer and intubation, he was eventually extubated [42].

To our knowledge, only three cases of anti-SARS-CoV-2 vaccination-induced MG have been described.

A review of immune-mediated diseases or flares within 28 days after SARS-CoV-2 mRNA vaccination from health care organizations in three countries (Israel, UK, USA) found only two cases of new onset MG following Pfizer BNT162b2 vaccination. Causality was assessed according to the WHO guidelines on surveillance of Adverse Events Following Immunisation (AEFI) [43]. One case presented 1 day after the second dose and promptly improved after steroid and PE. The second case presented 7 days after the second dose with ocular signs but worsened with bulbar and respiratory involvement requiring intubation. Overall, out of the 27 cases of immune-mediated AEFI, 25 received mRNA vaccines [44].

Another case report showed the case of a man who developed intermittent episodes of bulbar symptoms, including slurring speech and dysphagia, 4 weeks after the first dose and 2 days after the second dose of the Pfizer BNT162b2 anti-SARS-CoV-2 vaccine. Anti-AchR antibodies were markedly elevated and repetitive stimulation EMG showed decrement phenomenon, confirming the diagnosis of MG. Despite adequate treatment, he subsequently developed a severe generalised exacerbation, requiring hospitalisation, high-dose steroids, IVIg, intubation and PEG-tube placement, eventually followed by recovery and patient discharge [45].

Patone et al.’s population-based case series investigated hospital admissions from neurological complications occurring within 28 days after the first dose of anti-SARS-CoV-2 vaccination. The study found an increased risk of hospitalisation and death from myasthenic disorders in a window of 15–21 days after the first dose of ChAdOx1nCoV-19 vaccine (incidence rate ratio or IRR: 1.57). However, no association was identified with the BNT162b2 vaccine [46].

A recent Italian study investigating the safety of anti-SARS-CoV-2 vaccines in 104 myasthenic patients, found MG worsening cases after vaccination in 8/104 (7.7%, 7 after Pfizer BNT162b2 vaccine and 1 after Moderna mRNA-1273 vaccine), most of which were mild events. Remarkably, the frequency of disease exacerbation in anti-MuSK seropositive patients was much higher than other MG categories [47].

Two case reports concerning the immunogenicity of anti-SARS-CoV-2 vaccination and efficacy of revaccination in MG patients have been published. In the first one, a 74-year-old MG patient treated with mycophenolate, prednisone and eculizumab received both doses of the Pfizer BNT162b2, which failed to induce detectable specific circulating IgG or IFN-gamma T-cell responses. She was then administered both doses of Moderna M1273 vaccine, the aforementioned vaccine immunogenicity tests were repeated and resulted positive [48]. The second study regarded a 75-year-old MG patient under steroid and mycophenolate treatment, who received a complete cycle of the Moderna M1273 vaccine, resulting in the absence of detectable specific neutralising circulating antibodies, unlike immunocompetent individuals. Thus, the patient received two additional doses of the Pfizer BNT162b2 vaccine, after reducing mycophenolate dose 3 weeks prior to the first dose. In addition, he briefly interrupted both prednisone and mycophenolate intake the day before and for 3 consecutive days after the second dose. As a result, this second vaccination cycle elicited a strong humoral response, with an elevated titre of detectable neutralising antibodies [49].

We analysed a large cohort of 80 patients (41 males and 39 females), with a mean age of 60.4 years. None of our patients were treated with monoclonal antibodies, such as rituximab or eculizumab. Most of the patients were vaccinated with the Pfizer BNT162b2 (68/80 patients), five patients with the Moderna M1273 and three patients with Astrazeneca ChAdOx1 nCoV-19 vaccines. In 4 cases, it was not possible to identify the type of vaccine. We divided our cohort into 3 groups: 1) 69 patients were classified as stable and did not show any deterioration or exacerbation of MG symptoms; 2) 7 patients were treated with plasma-exchange or IVIg on a regular basis, making it difficult to identify any exacerbation of MG symptoms putatively related to the vaccine; 3) two patients (2.5%) experienced an MG exacerbation requiring steroid/immunosuppressant introduction or dose increase, within the chosen window period of 1 day—6 weeks from the second dose of vaccination. In particular, the time between the last vaccine dose and disease exacerbation was 1 and 15 days, respectively. Two patients (2.5%) had severe MG exacerbations requiring plasma-exchange or IVIg within the aforementioned window period. The time between the last vaccine dose and disease exacerbation was 7 and 10 days, respectively. Overall, 5% of patients developed MG exacerbation within the post-vaccine time window, all after the second dose of BNT162b2 vaccine. No exacerbations were recorded between the first and the second dose.

Thus, in our sample, four patients had a myasthenic exacerbation. Patient 1 developed a myasthenic crisis 7 days after the second dose, requiring both azathioprine introduction and PE, despite having already had mild disease worsening caused by a cystitis, earlier in the same month. Patient 2 developed a PE-requiring myasthenic crisis 10 days after the second dose, although he was concomitantly undergoing steroid tapering. During the previous month, the patient suffered from a similar crisis, associated with steroid dose reduction. Patients 3 and 4 had stable ocular MG but developed diplopia 1 and 15 days after the second dose, respectively. They fully recovered after steroid dose increase.

We also report the case of a 64-year-old healthy female subject who developed de novo anti-acetylcholine receptor antibody positive MG around 12 days after the second dose of vaccine.

## Discussion

### Safety

The safety of vaccines in MG is summarised in Table 2, where results are furtherly categorised by vaccine type. A survey has shown that opinions about the safety of vaccines in MG patients are variable [19]. Almost half of patient non-compliance cases were caused either by fear of myasthenic-exacerbations or by physicians not recommending the vaccine. In another study, an important factor influencing general practitioners’ indications was a history of myasthenic exacerbation following influenza vaccination. It is surprising that well-done case–control studies are so scarce and available only for influenza vaccination. However, the literature shows that influenza vaccine does not increase the risk of both clinical and serological myasthenic exacerbations, although some mild adverse effects, unrelated to MG, are possible. Such evidence was obtained through both retrospective and placebo-controlled prospective studies, which also demonstrated that these mild reactions were more frequent than in placebo-receiving subjects, yet not significantly. In addition, influenza-like illnesses have a higher potential risk of aggravating MG than influenza vaccination does. All of the abovementioned evidence leads to the conclusion that influenza vaccination is safe and its benefits outweigh the risks. Thus, it ought to be recommended to MG patients, as it might reduce the risk of upper respiratory tract infections and, consequently, of myasthenic exacerbations. Hence, a significant percentage of patient non-compliance to influenza vaccination could be overcome by both spreading these data to the neurologists and correctly informing MG patients about the safety of such prevention measure even with the help of patients’ associations.

**Table 2:** Safety of vaccines in patients affected by myasthenia gravis*

Vaccine	Authors	Number of patients	Study design	Follow-up period	Results/conclusions
Influenza	Bhaskar Roy et al. 2021 [19]	184 neurologists; 6465 MG pts, 2313 CIDP pts, 1907 GBS pts	Retrospective internet-based survey	None	Influenza vaccine was recommended by 82.6% of the respondent neurologists. A history of exacerbation following the influenza vaccine was the most important factor influencing vaccine recommendation
Influenza	Zinman et al. 2009 [20]	513 hospitalised MG pts	Retrospective, self-matched, population-based study	42 weeks	No significantly increased risk of hospitalization for exacerbation of MG following influenza vaccination. Mild exacerbations were not included
Influenza	Auriel et al. 2011 [21]	74 MG pts: 32 no vaccinated, 24 received H1N1 vaccine, 18 seasonal influenza vaccine, 20 both vaccines	Retrospective longitudinal, survey	At least 8 weeks	No exacerbations were found and only 3/24 patients who received H1N1 vaccine reported non-specific illnesses
Influenza	Hung Youl Seok et al. 2017 [22]	258 MG pts: 133 vaccinated versus, 121 influenza-like illness (ILI) or common cold	Retrospective multicenter survey	None	MG exacerbations occurred in 40% after ILI, in 15.6% after common colds, and 1.5% of vaccinated pts (p = 0.006). The risk was higher for ILI than for influenza vaccination
Influenza	Ellen Strijbos et al. 2019 [23]	47 anti-AchR AChR + MG pts: 29 with (IM +) and 18 without (IM-) immunosuppressive therapy; 47 healthy controls	Longitudinal prospective, placebo-controlled study	4 weeks	No clinical exacerbations nor significant change in AchR-antibodies titres after 4 weeks and between IM + and IM- after influenza vaccination
Influenza	Björn Tackenberg et al. 2018 [24]	62 MG pts: 31 vaccine vs 31 placebo	Longitudinal prospective, placebo-controlled trial	12 weeks	No significant difference in AchR-antibodies titres. Adverse events, exacerbations and therapeutical modifications were comparable between groups
Influenza	Wang et al. 2021 [25]	1 non-myasthenic subject	Case report	6 months	Onset of laryngeal MG 5 days after trivalent inactivated influenza vaccine. Significant attenuation of symptoms after 2 weeks of pyridostigmine and steroids
HBV	Domingo et al. 2009 [26]	1 asthmatic non-myasthenic subject and 1 MG patient	Case series	(not specified)	The non-myasthenic subject developed generalised anti-AchR + MG 1 week after HBV vaccine, while the MG patient had an exacerbation in the month following HBV vaccination
HBV	Biron et al. 1988 [28]	1 asthmatic non-myasthenic subject	Case report	(not specified)	Onset of generalised MG after general anaesthesia and HBV vaccine, treated with immunosuppressive drugs and plasma-exchange
HBV	Louzir et al. 2003 [29]	1 asthmatic non-myasthenic subject	Case report	(not specified)	Onset of MG after HBV vaccine; MG exacerbation, requiring ventilator care and IVIg, 4 years later
HBV	Shaw et al. 1988 [31]	41 reported neurologic adverse events in after plasma-derived HBV vaccination (from 1982 to 1985)	Post-marketing population-based case series	None	1 case of new-onset myasthenia gravis
HPV	Chung et al. 2018 [32]	1 non-myasthenic subject	Case report	5 months	After the second dose of HPV vaccine, the subject developed ocular and bulbar MG, with access to ICU. Complete resolution after 4 weeks
Japanese encephalitis virus (live attenuated vaccine)	Dan He et al. 2018 [33]	Retrospective: 4219 MG cases Prospective: 104 childhood-onset MG and 100 healthy controls	Retrospective, prospective longitudinal and experimental (mouse studies)	Retrospective: from 01/1982 to 07/2013 Prospective: from 01/2011 to 06/2013	Increase of anti-AchR-antibodies in both children and mice injected with LA-JEV; reduction of AchR density at NMJs, decreased muscle strength and response to repetitive nerve simulation in the same mice. Immunisation with a peptide of LA-JEV structurally similar to AchR induced a MG-like phenotype in mice, through molecular mimicry
Bacillus Calmette-Guerin	Takizawa et al. 2017 [34]	1 non-myasthenic subject with bladder cancer	Case report	1 year	Onset of ocular anti-AchR + MG after intravesical BCG. Complete resolution 12 days after steroid therapy
	Davalos et al. 2019 [35]	1 non-myasthenic subject with bladder cancer	Case report	17 months after BCG treatment	Onset of anti-AchR generalised MG within 1 month of intravesical BCG treatment. Treatments included steroids, IVIg and thymectomy; only minimal disease manifestations persisted
Tetanus	Strijbos et al. 2017 [36]	65 MG/LEMS pts (verum group), 23 MG pts (placebo group), 20 historical healthy controls	Longitudinal prospective, placebo-controlled	4 weeks	Anti-AChR, MuSK or VGCC titres remained unchanged 4 weeks after vaccination; moreover, no myasthenic exacerbations were registered. Tetanus revaccination is safe in MG/LEMS pts
Neisseria Meningitidis (meningococcus)	Tartof et al. [39]	327 children aged 2–10	Retrospective observational	6–12 months	Preliminary results showed 1 case of post-vaccine myasthenia gravis, subsequently refuted after physician investigator review
Staphylococcus pneumoniae (23 polysaccharidic antigen pneumococcal vaccine)	Nasca et al. 1990 [40]	25 MG pts, 11 control pts	Longitudinal prospective	28 ± 5 days	Only minor local side effects were reported; all MG patients remained clinically stable, without exacerbations after vaccination
SARS-CoV-2 (inactivated vaccine, adenovirus-vectored vaccine, recombinant subunit vaccine)	Ruan et al. 2021 [41]	22 MG pts	Retrospective, single-centre case series	4 weeks after vaccination; 18 months for patients with symptom worsening	2/22 patients had mild and promptly-resolving worsening of MG symptoms
SARS-CoV-2 (Moderna 1273 mRNA vaccine)	Tagliaferri et al. 2021 [42]	1 MG pt	Case report	(not specified)	Bulbar and respiratory MG crisis 1 week after second dose of Moderna vaccine, requiring ICU transfer and intubation
SARS-CoV-2 (Pfizer BNT162b2, Moderna 1273 mRNA and ChAdOx1 DNA vaccines)	Watad et al. 2021 [44]	27 subjects with immune-mediated diseases or flares within 28 days after SARS-CoV-2 vaccination	Review structured as a multi-centre case series	(not specified)	25/27 received mRNA vaccines One MG case 1 day after 2nd dose of Pfizer vaccine promptly improved after steroid and PE. The second case presented 7 days after the 2nd dose of Pfizer vaccine with ocular signs but worsened with bulbar and respiratory involvement requiring intubation
SARS-CoV-2 (Pfizer BNT162b2 mRNA vaccine)	Chavez et al. 2021 [45]	1 healthy subject	Case report	At least 4 months (not specified)	Onset of bulbar MG 2 days after the second dose of the Pfizer vaccine, requiring hospitalisation, PEG placement and intubation
SARS-CoV-2 (Pfizer BNT162b2 mRNA and ChAdOx1 DNA vaccines)	Patone et al. 2021 [46]	More than 32 million people residing in England (National Immunisation Management System database for SARS-CoV-2 vaccination and National Health System for COVID-19 data)	Population-based case series	28 days after the first dose of anti-SARS-CoV-2 vaccination or positive test	Increased risk of hospitalisation and death from myasthenic disorders in a window of 15–21 days after the first dose of ChAdOx1nCoV-19 vaccine (incidence rate ratio or IRR: 1.57)
SARS-CoV-2 (Pfizer BNT162b2 and Moderna 1273 mRNA vaccines)	Farina et al. 2022[47]	104 MG patients	Retrospective bicentric case series	4 weeks after a vaccine dose	MG worsening cases were found in 7.7% of pts

^*^Articles regarding safety of vaccinations in MG patients, found in the online database “PubMed”, are listed and summarised here. For each one, vaccine type, authors, number of patients included, study design, follow-up period and results/conclusions are indicated. Complete references are available in the “Bibliography” section

As far as the anti-tetanus revaccination is concerned, neither clinical nor serological MG exacerbations were reported. Similarly, the 23-antigen pneumococcal in MG patients only caused minor reactions at the injection site, without any cases of clinical exacerbation. The MenACWY-CRM vaccine against Neisseria meningitidis is equally safe as the only case of post-vaccine MG onset was refuted after physician investigator review.

Taking into account the previously mentioned evidence, together with the guidelines in use, patients undergoing chronic immunosuppressive therapies, including those affected by MG, should indeed receive inactivated and subunit vaccines. On the contrary, live attenuated vaccines are generally contraindicated, unless administered before the start of treatment regimens (at least 1 month for the anti-varicella vaccine). Such indications are dictated by the risk of reactivation of the viral strain used in these vaccines [50].

### Efficacy

The efficacy of vaccines in MG is summarised in *Table **3*. Regarding influenza vaccination, there are no statistically significant differences between MG patients and healthy subjects in the surrogate serological endpoint, namely the anti-influenza post-vaccination titres. Moreover, although azathioprine can theoretically reduce immune responses to vaccine, neither immunosuppressive medications nor thymectomy had a negative impact on vaccine efficacy. From the clinical point of view, results regarding vaccine efficacy are unclear. While one study found a statistically non-significant reduction of MG exacerbations after influenza-vaccine in a subset of older patients, another one found no differences between groups. In the latter study, the authors interpreted such findings as a consequence of the advanced mean age of the subjects included and the low global efficacy of the influenza vaccine during the years 2014–2015. Nevertheless, no studies have shown a significantly lower efficacy of such vaccine in the myasthenic population, compared to the general population.

**Table 3:** Efficacy of vaccines in patients affected by myasthenia gravis*

Vaccine	Authors	Number of patients	Study design	Follow-up period	Results / Conclusions
Influenza	Zinman et al. 2009 [20]	513 hospitalised MG pts	Retrospective, self-matched, population-based study	42 weeks	Older pts had lower risk for exacerbation of MG in the immediate post-vaccination period, yet this was not statistically significant (*p* = 0.073)
Influenza	Hung Youl Seok et al. 2017 [22]	258 MG pts: 133 received influenza vaccination; 121 experienced a common cold or an influenza-like illness (ILI)	Retrospective multicentre survey	None	The vaccine does not confer protection from ILI: difference between 11/133 (8.3%) vaccinated pts with ILI and 14/125 (11.2%) of the unvaccinated pts with ILI was not significant
Influenza	Ellen Strijbos et al. 2019 [23]	47 anti-AchR AChR + MG pts: 29 with (IM +) and 18 without (IM-) immunosuppressive therapy; 47 healthy controls	Longitudinal prospective, placebo-controlled study	4 weeks	Post-vaccination seroprotection was similar between healthy controls, IM + and IM- and between thymectomised and non-thymectomised patients
Tetanus	Strijbos et al. 2017 [36]	65 MG/LEMS pts (verum group), 23 anti-AchR + MG pts (placebo group), 20 historical healthy controls	Longitudinal prospective, Placebo-controlled	4 weeks	MG pts treated with immunosuppressants had significantly lower pre- and post-vaccine titres compared to controls (*p* < 0.01; *p* < 0.01). Although 5 pts were non responders, all patients had protective pre- and post-vaccine titres. Tetanus revaccination is thus effective in MG/LEMS pts
Tetanus	Csuka et al. 2013 [38]	158 MG pts, 279 systemic lupus erythematosus (SLE) pts and 208 healthy control subjects	Case–control study	None	Tetanus-antitoxoid IgG titre decreased only in the elderly subjects in all 3 groups and was not associated with the presence of anti-AchR in MG pts (*p* = 0.112). Vaccine-induced immunity was comparable to that of the general population
Diphtheria	Csuka et al. 2013 [38]	158 MG patients, 279 systemic lupus erythematosus (SLE) patients and 208 healthy control subjects	Case–control study	None	Diphtheria-antitoxin IgG titre decreased significantly (*p* < 0.001) with age in all 3 groups and was not associated with the presence of anti-AchR in MG patients (*p* = 0.196). Vaccine-induced immunity was comparable to that of the general population
Staphylococcus pneumoniae (23 polysaccharidic antigen pneumococcal vaccine)	Nasca et al. 1990 [40]	25 MG pts (5 subgroups), 11 control pts, 17 healthy volunteers	Longitudinal prospective	28 ± 5 days	Post-immunization titres were not significantly different between MG and control patients. MG patients receiving prednisone and chronic plasmapheresis had higher pre-immunization titres than did other patient groups and had significantly higher postimmunization titres than other MG subgroups. Antibody rebound and overshoot phenomena after plasmapheresis is a possible explanation
SARS-CoV2 (Pfizer mRNA BNT162b2 and Moderna mRNA 1273 vaccines)	Plymate et al. [48]	1 MG pts	Case report	Immunogenicity tests 71 days after end of 1^st^ cycle, 85 days after end of 2^nd^ cycle	No detectable specific T- and B-cells vaccine immunogenicity tests after 1st cycle with BNT162b2; positive tests after 2nd cycle with M1273. Repeat vaccination might represent a strategy to increase vaccine efficacy
SARS-CoV2 (Pfizer mRNA BNT162b2 and Moderna mRNA 1273 vaccines)	Golding et al. [49]	1 MG pts	Case report	Immunogenicity tests 4 weeks after end of 1^st^ cycle, 2 weeks after end of 2^nd^ cycle	No detectable specific neutralising antibodies after 1st cycle with M1273; elevated titre after 2nd cycle with Pfizer BNT162b2 and temporary interruption of immunosuppressive therapy. Although revaccination might have played a role, transitory remodulation of immunosuppressive therapies may represent a strategy to increase vaccine efficacy

^*^Articles regarding safety of vaccinations in MG patients, found in the online database “PubMed”, are listed and summarised here. For each one, vaccine type, authors, number of patients included, study design, follow-up period and results/conclusions are indicated. Complete references are available in the “Bibliography” section

Regarding other vaccines, post-vaccine titres after tetanus and diphtheria vaccination are not influenced by anti-AchR antibodies levels and can be either equal (tetanus) or lower (tetanus and diphtheria) than those of healthy subjects. In the latter case, however, antibodies levels are still protective, thus vaccine-induced immunity in MG patients is comparable to that of the general population.

The only study investigating the impact of pneumococcal vaccine on MG patients showed that patients not undergoing immunotherapy or those receiving prednisone have pre- and post-immunisation titres similar to those of control patients. On the other hand, plasmapheresis seems to induce an overshooting phenomenon, namely an increase in antibody titres, which, in turn, is abolished in case of concomitant azathioprine treatment.

### New onset post-vaccine myasthenia gravis

Several cases of new onset post-vaccine MG have been reported. Three out of the four cases due to HBV vaccination regarded asthmatic patients that received obsolete human donor plasma-derived vaccines. Nonetheless, a comprehensive review deemed post-HBV-vaccine MG as extremely rare [30]. There were two case reports of MG after intravesical Bacillus Calmette-Guerin administration for bladder cancer, although no cases were reported when used as anti-tubercular vaccine. In addition, just one case of new onset post-HPV-vaccine MG was reported. Remarkably, three cases of new onset MG were reported in association with the anti-SARS-CoV-2 Pfizer BNT162b2. In two of these, the clinical course was severe. Lastly, we report a fourth case, which was characterised by mild course.

Furthermore, an interesting finding is that live-attenuated Japanese encephalitis virus vaccination (LA-JEV) has been proposed as a potential risk factor for childhood-onset MG, in countries where such vaccination is routine. As a matter of fact, in both humans and mice, LA-JEV elicited a significant serologic rise of anti-AchR antibodies. The latter discovery, together with the evidence of post-vaccine MG-like illness in mice and the cross-reactivity between LA-JEV and the acetylcholine receptor, led to the conclusion that further studies are needed to corroborate these data in humans and that LA-JEV should probably be administered more cautiously in children.

We suggest keeping in mind the possibility of new onset MG, even if this seems a rare event and not frequently associated with vaccines. Thus, in case a patient manifests muscle fatigability and fluctuating weakness after one of these vaccines, it is important to promptly start MG diagnostic work-up and adequate therapy.

### SARS-CoV-2

To date, since the approval of anti-SARS-CoV-2 vaccines across the globe, only few studies have focused on its effects on MG patients. The published literature suggests that it is safe in this patient population, as only few cases of vaccine-related exacerbation, within a plausible pathogenetic window, have been reported. Among those previously reported, two were mild exacerbations, included in a retrospective case-series conducted on 22 MG patients (9%) and they seemed unrelated to pre-vaccine disease severity. Another one was a case report of a severe myasthenic worsening.

In our large cohort, we found an exacerbation of MG that required an increase in the steroid or immunosuppression with prompt control of the symptoms in 2 out of 80 patients (2.5%) while in other 2 patients (2.5%) we recorded a myasthenic crisis or bulbar exacerbation requiring plasma-exchange or IVIg. All cases occurred after the second dose of Pfizer BNT162b2 vaccine. In line with Patone et al. [46], we did not find cases of myasthenic exacerbations between the first and second dose, as only three of patients included received ChAdOx1 nCoV-19 vaccine. Our results are very similar to a recent paper by Farina et al. that found myasthenic worsening cases in 7.7% of a large group of vaccinated Italian MG patients [47].

These preliminary data suggest that SARS-CoV-2 vaccine benefits outweigh the risks in MG patients, even if exacerbation of MG symptoms with variable severity could be present in up to 8–9% of the cases. This might be an overestimation, considering that in a large cohort of MG patients around 5% of patients are found symptomatic at each follow-up examination [51]. Moreover, as we noted in our sample, confounding factors can be a recent dose reduction in immunosuppressive therapy or chronic treatment with plasma-exchange or IVIg. EMA guidelines suggest that IVIg can impair the efficacy of live attenuated vaccines for up to 3 months, thus also the time between IVIg administration and a dose of vaccine might represent a confounder worth considering [52]. Another aspect to acknowledge is that worsening in unstable patients, usually in the first year from onset, is difficult to correlate with vaccines. Thus, additional case–control prospective studies are indeed necessary to precisely assess the entity of the exacerbation risk and the presence of predisposing factors.

On the other hand, it is worth remembering that COVID-19 is a severe respiratory and systemic disease. There is currently controversial data regarding the relationship between COVID-19 and MG outcome, due to the coexistence of reports of both stable[53] and worsening[46, 54–57] MG cases, during the course of COVID-19. In any case, available data suggest that there is no association between COVID-19 severity and risk of myasthenic exacerbation, as even mild COVID-19 cases may trigger MG worsening [58]. In parallel, an important French study found that higher baseline MG severity was associated with COVID-19 severity, whereas neither of the latter two were associated with MG outcome after COVID-19 [59].

Concerning adverse effects as a whole, a study comparing medium-term (within 1 month) adverse reactions induced by first and second doses of the mRNA Pfizer BNT162b2 vaccine shows that main adverse events (AE) reported were non-serious, mild systemic reactions. Remarkably, not only were they more frequent after the second dose, but also after the first dose of patients previously affected by COVID-19 [60]. The putative underlying reason was attributed to the more intense systemic immune response that characterises these conditions, in accordance to anti-SARS-CoV-2 antibody-response studies [61–64]. Thus, it would be advisable to carefully monitor for immune reactions among these subjects.

Regarding anti-SARS-CoV-2 vaccination efficacy in MG patients, the published literature suggests that repeat vaccination might represent a strategy to increase vaccine efficacy, through putative repeated antigen exposure mechanisms. Furthermore, transitory remodulation or interruption of immunosuppressive therapy may lead to increased vaccine efficacy, however with potentially increased risk of worsening of the underlying disease.

## Conclusions

The review of the literature suggests that inactivated and subunit vaccines are safe in myasthenic patients. In addition, neither clinical nor serological differences in efficacy have been reported between the latter ones and the general population; hence, these vaccinations should be recommended and administered to myasthenic patients. Remarkably, increasing vaccination rates against respiratory pathogens might reduce the burden of disease exacerbation at the population level. Regarding anti-SARS-CoV-2 vaccination, results from our study and from available literature suggest that it might rarely lead to disease worsening. However, the evidence has shown that the combination of MG and COVID-19 might trigger the exacerbation of either of the two. Thus, given the entity of the current pandemics, we believe that the benefits of anti-SARS-CoV-2 vaccination in myasthenic patients still outweigh by far the potential risks. In addition, the reported cases of new onset post-vaccine MG seem to be mostly anecdotal, except those putatively related to the Japanese encephalitis vaccination, which are indeed worth investigating.
